# Shifting Narratives in Media Coverage Across a Decade of Drug Discourse in the Philadelphia Inquirer: Qualitative Sentiment Analysis

**DOI:** 10.2196/56004

**Published:** 2025-05-13

**Authors:** Layla Bouzoubaa, Ramtin Ehsani, Preetha Chatterjee, Rezvaneh Rezapour

**Affiliations:** 1 College of Computing & Informatics Drexel University Philadelphia, PA United States

**Keywords:** natural language processing, drug use, media analysis, sentiment analysis, infodemiology, news

## Abstract

**Background:**

The media has immense power in shaping public narratives surrounding sensitive topics such as substance use. Its portrayals can unintentionally fuel harmful stereotypes and stigma, negatively impacting individuals struggling with addiction, influencing policy decisions, and hindering broader public health efforts.

**Objective:**

This study aimed to examine how the regional newspaper, The Philadelphia Inquirer, covered events related to illicit drug use between 2013 and 2022, focusing on linguistic patterns and themes associated with specific types of substances.

**Methods:**

We collected a dataset of 157,476 articles published in The Philadelphia Inquirer between 2013 and 2022 and categorized mentioned substances into 8 classes: stimulants, narcotics, cannabis, hallucinogens, depressants, designer drugs, drugs of concern, and treatment medications. From these 157,476 articles, we identified 3661 (2.32%) that mentioned at least 1 substance with potential for misuse. Using dynamic topic modeling, we analyzed thematic evolution in coverage across different drug classes. We then applied aspect-based sentiment analysis to extract the most significant phrases mentioned in each distinct drug class annually and examined the sentiments around these aspects to understand shifting discourse patterns.

**Results:**

Cannabis (1575/3661, 43.02%) and narcotics (1361/3661, 37.17%) dominated the coverage, with 2018 showing peak drug-related reporting (666/3661, 18.19%). Our substance co-occurrence analysis revealed that heroin was most frequently discussed alongside treatment medications (methadone, naloxone, and buprenorphine), reflecting evolving approaches to opioid use disorder. Topic modeling revealed distinct themes across drug classes: legislative and medical aspects dominated cannabis coverage, while narcotics coverage focused heavily on overdose deaths and safe injection sites, particularly during 2017 to 2018. Stimulant coverage centered on feature news and crime-related reporting, while treatment coverage showed an increasing focus on overdose prevention by 2021. The aspect-based sentiment analysis showed that 74.3% (165/222) of extracted aspects were portrayed negatively across all drug classes, with narcotics maintaining consistently negative sentiment throughout the period. However, some drug classes showed notable evolution: hallucinogens demonstrated a marked shift in sentiment score (SS) from negative coverage in 2013 (–0.79 SS) to positive coverage of therapeutic applications by 2021 (+0.47 SS), while cannabis coverage reflected complex societal debates, with industry and business aspects showing strong positive sentiment score peaks (0.64 SS in 2019) even as legislation and policy aspects remained volatile (–0.76 SS in 2013 to 0.61 SS in 2019 and declining to –0.31 SS by 2022).

**Conclusions:**

Our analysis revealed a predominance of negative and punitive language in drug-related news coverage, with limited representation of harm reduction principles. While some drug classes, particularly cannabis and hallucinogens, saw evolving narratives toward medical applications and policy reform, coverage of narcotics remained primarily focused on crime and overdose. These findings suggest a need for more balanced reporting that incorporates harm reduction perspectives and avoids potentially stigmatizing language when covering substance use disorders.

## Introduction

### Background

The relationship between media representation and public perception is a complex interplay that has profound implications for societal attitudes and policies [1-3]. Media, as a primary source of information for many, has the power to shape, reinforce, or challenge societal norms and beliefs [4,5]. When it comes to issues of public health, such as substance use disorder (SUD), the media’s portrayal can either support evidence-based interventions or perpetuate misconceptions and stigmas [6].

Previous research underscores the media’s powerful role in shaping perceptions about drugs and issues related to SUD [7-10]. For instance, in the late 1990s in Australia, a program aimed at controlling heroin-related issues faced political defeat, largely due to the media’s negative portrayal of heroin users as “deviants” [11]. Similarly, exaggerated media narratives around bath salts overshadowed clinical studies, leading to their prohibition [12,13]. Research by Denham [14] further highlights the media’s propensity to motivate moral panics about drugs, even when actual use rates remain stable [14].

Moreover, the media’s portrayal of stigmatized subjects, such as illicit drug use, can lead to stigma, discrimination, and reluctance to seek treatment [15,16]. For example, the media often emphasizes punitive measures against users and dealers, potentially influencing public attitudes and behaviors [17-19]. Caburnay et al [16] also found that media coverage can sway individual health behaviors, suggesting its potential impact on attitudes toward drug use.

Harm reduction, a public health strategy, seeks to mitigate the adverse effects of drug use without mandating complete cessation [20]. However, media coverage often lacks a comprehensive view of this approach. A study on Canadian news outlets revealed a tendency to focus on singular, controversial harm reduction strategies [21]. However, when conveyed appropriately, harm reduction messages can significantly diminish the societal repercussions of drug use, especially when they resonate with the audience’s values and present a holistic solution [22]. These examples show the importance of critically assessing media representations of substance use, ensuring that they are both accurate and holistic.

### Study Rationale and Aims

The pervasive issue of illicit drug use and its associated consequences has been a topic of concern in many urban areas across the United States. The city of Philadelphia has been at the forefront of this crisis, dealing with the devastating effects of drug addiction and its ripple effects on the community. Philadelphia has the second highest rate of overdose deaths (71 per 100,000) in the country [23] and is commonly known as one of the epicenters of the “opioid epidemic” in the United States [24]. In 2021, there were >1250 unintentional drug overdose deaths in Philadelphia [25], the highest number on record. Most of these deaths were caused by opioids, such as heroin and fentanyl [26]. Despite the city’s efforts to address the problem, such as expanding access to social services in the hardest-hit neighborhoods, several challenges remain, including the lack of affordable housing and the stigma associated with drug use and SUDs [27]. These challenges can make it difficult for people to seek help [28].

Given Philadelphia’s position at the epicenter of the US drug crisis, examining local media coverage becomes crucial for understanding how drug-related issues are presented to the public. The Philadelphia Inquirer, established in 1829, is the city’s largest newspaper, with a daily circulation of >100,000 readers, and serves as a primary source of local news coverage. As the third oldest surviving daily newspaper in the United States, it has historically provided extensive coverage of the city’s social issues, including public health challenges such as substance use. This study analyzed a decade of coverage (2013 to 2022) from The Philadelphia Inquirer, a period that encompasses significant shifts in drug policy and public health approaches, including the rise of the opioid crisis, the emergence of fentanyl as a dominant threat, and evolving attitudes toward cannabis legalization. This time frame allows us to capture both the intensification of the opioid crisis and the evolution of public health responses to substance use. Our study aimed to understand how The Philadelphia Inquirer’s coverage of illicit substances has evolved over time, with particular attention to their framing and contextual portrayal. Specifically, our study addresses 3 research questions (RQs):

- RQ1: How have the characteristics and nuances of mentions related to commonly abused substances evolved over the decade?
- RQ2: In the context of these substances, which overarching themes and subtopics have dominated the discourse, and how have they shifted over time?
- RQ3: What are the multifaceted dimensions, sentiments, and narratives associated with the portrayal of commonly abused substances?

Through this analysis, we seek to understand how media coverage may influence public perception and policy responses to substance use in a major urban center particularly affected by the ongoing drug crisis.

### Prior Work

Few studies have investigated the media coverage of illicit drugs and their change over time, particularly in the United States [15,16,29]. A recent study [7] investigating the coverage of illicit drug use by a Canadian newspaper found that it focused on basic social representations, such as attribution of the responsibility for the opioid crisis to a few collectives, including pharmaceutical companies and physicians, or the overall drug supply, and that a shift to less stigmatizing language could positively influence public perception. The study suggests that closer collaboration between the media and the research community is needed to achieve a better understanding of the issue. Ketamine, a psychoactive drug that has attained positive outcomes in treating severe, treatment-resistant depression, was found to be most associated with themes of abuse, legality, and clinical utility as an anesthetic when it was reported in North American news outlets between 2000 and 2015 [30]. This finding suggests that changes in news media reporting could influence how substances, such as ketamine, are received as viable treatment options, and that guidance is required for journalists on objective reporting of medical research findings.

Google News Archives and cause-of-death records published by the National Center for Health Statistics between 1999 and 2005 were explored for patterns in mentions of the opioid epidemic, and a noteworthy correlation was discovered between the number of news articles and the rates of opioid-related overdose deaths over time [31].

To comprehend the dissimilarities and similarities of media coverage based on drug types, Hayden Griffin et al [19] analyzed 487 news articles published in a national media outlet in Malaysia over a 2-year period. They found that amphetamines, opiates, and cannabis received most of the media coverage, and the discussion of these drugs was primarily in relation to criminal justice. Similarly, Hughes et al [18] conducted a framing analysis of Australian news media coverage of illicit drugs between 2003 and 2008. The study found that criminal justice topics are dominant, but nonlegal issues are also highlighted. The media frames can differ between drugs, with amphetamines portrayed most negatively and cocaine most neutrally. Their findings suggest that sensationalized reporting of drug events is more prevalent during specific episodes and may not be representative of the norm. Our findings do not indicate that The Philadelphia Inquirer uses tactics that sensationalize the current state of drug use in Philadelphia; however, there is a lack of messaging framed around harm reductionist principles or that normalizes evidence-based approaches to substance use.

## Methods

### Data Collection and Substance Classification

We collected 157,476 news articles from The Philadelphia Inquirer from January 1, 2013, to December 31, 2022, using the *ProQuest* database platform. Each article in our dataset comprises the complete text along with metadata attributes, such as publication date, author, title, links, and subject keywords.

### Drug Class Assignment

We compiled our list of substances using the Commonly Used Drugs Charts from the National Institute on Drug Abuse [32], which provides an overview of frequently used legal and illegal drugs. We then classified these substances according to the categories established by the Drug Enforcement Administration [33]. To ensure accuracy in our classifications, we manually reviewed and removed ambiguous terms with multiple meanings (eg, the term “pot” could refer to “flowering pot” or “cooking pot” in addition to marijuana). The Drug Enforcement Administration classifies drugs into 9 categories:

1. Cannabis: marijuana is a mind-altering (psychoactive) drug produced by the cannabis sativa plant. “Cannabis” and “marijuana” are often used interchangeably, resulting in the appearance of cannabis as both the drug class and the drug name.
2. Depressants: these are known to induce sleep, relieve anxiety and muscle spasms, and prevent seizures (eg, barbiturates and sedative-hypnotic substances, such as gamma-hydroxybutyrate).
3. Designer drugs: these are produced illicitly with a slightly altered chemical structure to mimic the pharmacological effects of controlled substances (eg, synthetic marijuana or synthetic cathinones).
4. Drugs of concern: these are unregulated drugs that can be harmful if abused (eg, kratom and xylazine).
5. Hallucinogens: these are derived from plants and fungi and renowned for their capacity to modify human perception and mood (eg, lysergic acid diethylamide, mushrooms, and ecstasy).
6. Narcotics: these refer to opium, opium derivatives, and their semisynthetic substitutes. “Opioid” is a more current and precise term to describe these drugs (eg, heroin, OxyContin, codeine, morphine, and fentanyl).
7. Stimulants: these are drugs that accelerate the body’s functions (eg, methamphetamine, cocaine, and amphetamines).
8. Treatment: these are substances aiding the treatment of opioid addiction (eg, methadone, Suboxone, and naloxone).
9. Miscellaneous: these are substances that can be abused but do not belong to any class (eg, steroids).

Using our mapped list of drug names, we identified news articles that mention any of these drugs in either their title or text. Subsequently, we grouped these articles into clusters based on the respective drug classes. If an article mentioned drugs from multiple classes, we assigned it to all relevant drug classes.

### Topic Modeling

To investigate how drug-related topics evolved over time in news coverage, we used dynamic topic modeling [34] using bidirectional encoder representations from transformers topic modeling technique (BERTopic) [35], an advanced method that identifies themes in a corpus of texts. Unlike traditional approaches, such as latent Dirichlet allocation, BERTopic better captures the context and meaning of words, producing more coherent and interpretable results [35]. We analyzed each drug class separately to understand how the coverage of different substances changed over time. Before topic modeling, we implemented several text preprocessing steps to improve the quality of our analysis. We standardized the text by converting all characters to lowercase and expanding contractions to their full form (eg, “don't” to “do not”). We then removed elements that could interfere with the analysis, such as URLs and common stop words (eg, “the,” “and,” as well as “or”) that do not carry significant meaning. These preprocessing steps reduced noise in the data and improved the model’s ability to identify meaningful patterns and topics. A technical limitation of our analysis with bidirectional encoder representations from transformers (BERT) is its maximum token limit of 512 tokens, while the median length of our articles was 749 (SD 573; IQR 511-1014) tokens. However, this limitation aligned well with journalistic writing conventions. News articles typically follow the inverted pyramid style [36], in which the most newsworthy information appears at the beginning of an article, followed by supporting details. This structure allowed us to confidently use the first 512 tokens of each article, as they typically contained the most salient information and key themes of the full text. This approach allowed us to work within the token limit and retain the essential content for our analysis.

To ensure high-quality results, we refined our analysis through multiple iterations following established topic modeling validation approaches [37,38]. We set specific parameters to optimize topic coherence, including capping the top number of words per topic at 10, and looked for words and phrases that commonly appear together (ie, n-grams) to provide richer context. We evaluated the quality of our results using 3 criteria: topic coherence (measured by a coherence score with a threshold >0.5), topic distinctiveness (minimal overlap in the top 10 keywords between topics), and human interpretability (clear thematic focus discernible from top keywords) [39]. Through iterative refinement, we systematically reduced the number of topics to 4 per drug class based on achieving maximum coherence scores, minimal thematic overlap, and clear interpretability [40].

To characterize and validate the topics generated by BERTopic, we conducted a detailed analysis of the representative articles. For each topic identified, 2 researchers independently examined the top 20 articles with the highest topic representation scores, noting common themes and key narratives. This human analysis helped us understand how the algorithmically derived topics appeared in actual news coverage and enabled us to develop more meaningful topic labels. For instance, when examining articles highly associated with what BERTopic initially labeled as “law enforcement” within the cannabis class, our review revealed a more nuanced focus on legislative processes and policy reform, leading us to refine this topic’s characterization. Disagreements in topic interpretation were resolved through discussion with a third researcher until a consensus was reached. This process of combining computational topic modeling with human analysis of representative articles enhanced our understanding of the thematic evolution in drug-related coverage.

### Aspect-Based Sentiment Analysis

We used aspect-based sentiment analysis rather than traditional sentiment analysis because drug-related articles often contain multiple viewpoints and sentiments within the same text. While traditional sentiment analysis assigns a single sentiment score to an entire document, aspect-based sentiment analysis allows us to capture nuanced sentiments associated with specific aspects of drug discourse, for instance, distinguishing between sentiments about law enforcement approaches versus public health initiatives, even within the same article. To extract these aspects from the articles, we used key phrase extraction to identify words and phrases with the highest significance and relevance within each news article. As key phrases encapsulate the essence of an entire document, they serve as essential tools for retrieving critical information from large and diverse datasets [41].

While various tools and techniques are available for key phrase extraction from documents, it is notable that most of these models generally focus on the statistical properties of text rather than semantic similarity [42]. We selected KeyBERT [42] for phrase extraction because of its ability to capture context and identify meaningful phrases. The model combines 3 key components: the TextRank algorithm [43] (which ranks phrases based on their importance within the text), BERT embeddings [44] (numerical representations that capture the meaning of words and phrases), and cosine similarity [42] (a measure of how closely related 2 pieces of text are). To improve accuracy, we used vectorizers (tools that convert text into structured patterns) that analyze grammatical patterns to ensure that extracted phrases were linguistically meaningful. We analyzed articles within each drug class year by year from 2013 to 2022, allowing us to track how key phrases and their associated topics evolved over time. For each article, we compared the extracted phrases with the overall article content using cosine similarity scores, retaining only those phrases that reached a similarity threshold of 40%. We determined this threshold through careful testing, finding that it effectively balanced between capturing relevant phrases and excluding irrelevant or redundant information.

To analyze sentiment, we first located all paragraphs containing each identified aspect, allowing us to focus on relevant content and reduce noise. We then used the NewSentiment model [45], which is specifically designed for analyzing news content, to determine whether the discussion of each aspect was positive, negative, or neutral. This model was pretrained on a large dataset of political news articles [45], making it well suited for analyzing journalistic writing. For each aspect, we calculated a normalized sentiment score (SS) by subtracting the negative sentiment probability from the positive sentiment probability and dividing by their sum, resulting in scores ranging from –1 (most negative) to 1 (most positive). To facilitate interpretation and identify broader patterns, we manually grouped semantically similar aspects into thematic categories for each drug class (eg, “Legislation/Policy,” “Medical Use” for cannabis; “Overdose Prevention,” “Opioid Treatment” for treatment drugs). We then calculated mean sentiment scores and CIs for each aspect group per year, allowing us to track how sentiment toward different themes evolved over time.

### Ethical Considerations

This study analyzed publicly available news articles from The Philadelphia Inquirer accessed through the ProQuest database platform. No data involving human participants were collected, analyzed, or stored during this research. All article data were handled in aggregate form, and no personally identifiable information was extracted or analyzed.

## Results

### Substance Distribution and Co-Occurrence Patterns

Of the 157,476 articles analyzed, 3661 (2.32%) referenced at least one drug class. The distribution of these articles across drug classes and years is shown in Table 1. We observed a steady increase in explicit substance mentions, followed by a decline (COVID-19 pandemic effect, presumably around 2020) in The Philadelphia Inquirer over the past decade. Cannabis was the most frequently discussed drug class, followed by narcotics (ie, opioids). The annual count of news articles indicates that 2018 saw the highest frequency of drug-related mentions. Narcotics were the second most discussed drug class and the most prevalent in 2017 and 2018, suggesting that the opioid crisis was a major focus of news coverage during those years.

**Figure 1:**
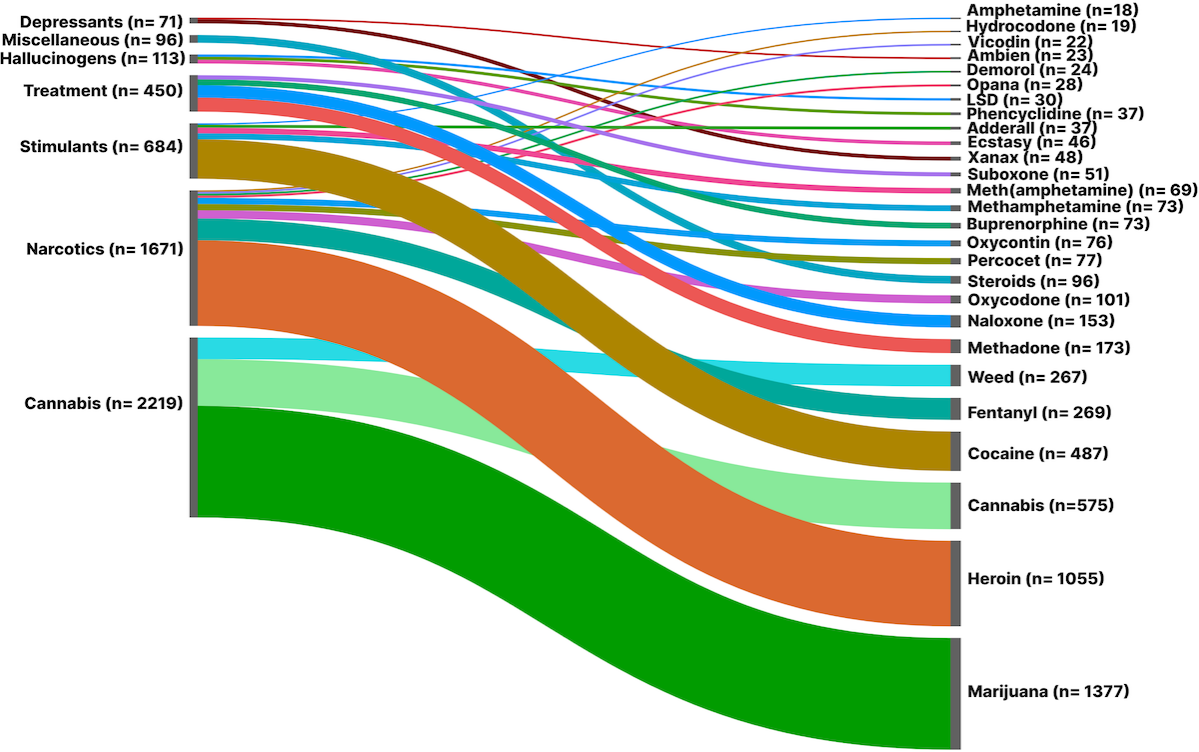
Number of substances per drug class in our dataset. LSD: lysergic acid diethylamide.

Further analysis of drug mentions by class shows that cannabis is primarily associated with marijuana (Figure 1). Despite the controversial origins of the term “marijuana” [46], it has been consistently used in news articles over the years. Among the depressant drugs, Xanax and Ambien were the most frequently mentioned, likely due to their widespread use for anxiety and sleep disorders and their potential for abuse. Similarly, cocaine and ecstasy were the most frequently mentioned drugs in the stimulant and hallucinogen drug classes, respectively, indicating their enduring popularity.

**Table 1:** Distribution of articles per drug classes by year.

Year	Cannabis (n=1575, 43.02%)	Depressants (n=106, 2.89%)	DD^a^ (n=16, 0.43%)	DC^b^ (n=11, 0.3%)	Hallucinogens (n=137, 3.74%)	Narcotics (n=1361, 37.17%)	Stimulants (n=627, 17.13%)	Treatment (n=449, 12.26%)	Total^c^ (n=3661, 100%)
2013	138 (8.76)	23 (21.7)	3 (18.75)	2 (18.18)	20 (14.6)	*142*^d^ (10.43)	108 (17.22)	16 (3.56)	452 (100)
2014	*146 (9.27)*	10 (9.43)	0 (0)	1 (9.1)	10 (7.3)	117 (8.6)	64 (10.2)	21 (4.68)	369 (100)
2015	*122 (7.75)*	6 (5.66)	0 (0)	0 (0)	15 (10.95)	117 (8.6)	49 (7.8)	30 (6.68)	339 (100)
2016	*150 (9.52)*	12 (11.32)	0 (0)	0 (0)	19 (13.86)	132 (9.7)	63 (10.05)	43 (9.58)	419 (100)
2017	194 (12.32)	12 (11.32)	0 (0)	0 (0)	9 (6.57)	*223 (16.39)*	53 (8.45)	78 (17.37)	569 (100)
2018	221 (14.03)	14 (13.2)	8 (50)	6 (54.54)	18 (13.14)	*228 (16.75)*	73 (11.64)	98 (21.82)	666 (100)
2019	*231 (14.67)*	10 (9.43)	4 (25)	2 (18.18)	11 (8.03)	148 (10.87)	90 (14.35)	48 (10.69)	544 (100)
2020	*113 (7.17)*	7 (6.6)	0 (0)	0 (0)	7 (5.1)	69 (5.07)	48 (7.67)	31 (6.9)	275 (100)
2021	*118 (7.49)*	7 (6.6)	0 (0)	0 (0)	16 (11.68)	86 (6.32)	41 (6.54)	41 (9.13)	309 (100)
2022	*142 (9.02)*	5 (4.72)	1 (6.25)	0 (0)	12 (8.76)	99 (7.27)	38 (6.06)	43 (9.58)	340 (100)
Total	1575 (100)	106 (100)	16 (100)	11 (100)	137 (100)	1361 (100)	627 (100)	449 (100)	—^e^

^a^DD: designer drug.

^b^DC: drug of concern.

^c^The total sum of all drug class mentions exceeds the number of unique articles containing substance mentions (n=3661) because individual articles may mention substances from multiple drug classes.

^d^Italicization indicates the most frequent drug class.

^e^Not applicable.

Our co-occurrence analysis revealed an association between the frequency with which certain substances are discussed and provisional overdose and use patterns. Table 2 shows that heroin, one of the most prevalent substances in Philadelphia, was overtaken by fentanyl in 2018, buprenorphine in 2021, and buprenorphine and fentanyl in 2022. Furthermore, our analysis indicated that narcotics and treatment drugs, such as methadone, naloxone, and buprenorphine, were the second most commonly colocated drugs in articles. Treatment drugs such as methadone, naloxone, and buprenorphine are viewed as positive and proactive responses to opioid use disorder (OUD) [47]. Buprenorphine, a safe and effective treatment for OUD, emerged in 2020 alongside heroin, followed by cannabis in 2021 and 2022.

**Table 2:** Most frequently co-occurring drugs by year.

Year and drugs		Value, n (%)
**2013 (n=452)**		
	Heroin and methadone	52 (11.5)
	Heroin and cocaine	45 (10)
	Barbiturates and cannabis	16 (3.5)
**2014 (n=369)**		
	Heroin and naloxone	42 (11.4)
	Marijuana and cocaine	36 (9.8)
	Heroin and cocaine	34 (9.2)
**2015 (n=339)**		
	Heroin and marijuana	40 (11.8)
	Heroin and methadone	19 (5.6)
	Heroin and methamphetamine	11 (3.2)
**2016 (n=419)**		
	Heroin and marijuana	51 (12.2)
	Heroin and cocaine	45 (10.7)
	Heroin and naloxone	44 (10.5)
**2017 (n=569)**		
	Heroin and methadone	157 (27.6)
	Heroin and cocaine	90 (15.8)
	Heroin and naloxone	80 (14.1)
**2018 (n=666)**		
	Fentanyl and cocaine	270 (40.5)
	Heroin and cocaine	194 (29.1)
	Heroin and marijuana	127 (19.1)
**2019 (n=544)**		
	Heroin and marijuana	115 (21.2)
	Heroin and naloxone	77 (14.2)
	Heroin and cocaine	71 (13.1)
**2020 (n=275)**		
	Heroin and methadone	198 (72)
	Heroin and buprenorphine	81 (29.5)
	Fentanyl and cocaine	53 (19.3)
**2021 (n=309)**		
	Buprenorphine and cannabis	108 (35)
	Heroin and cannabis	78 (25.2)
	Heroin and marijuana	41 (13.3)
**2022 (n=340)**		
	Buprenorphine and cannabis	351 (103.2^a^)
	Buprenorphine and marijuana	153 (45)
	Fentanyl and cocaine	114 (33.5)

^a^Percentages are calculated relative to the total number of drug-mentioning articles that year. Values may exceed 100% as substances can co-occur multiple times within individual articles.

### Thematic Evolution by Drug Class

**Figure 2:**
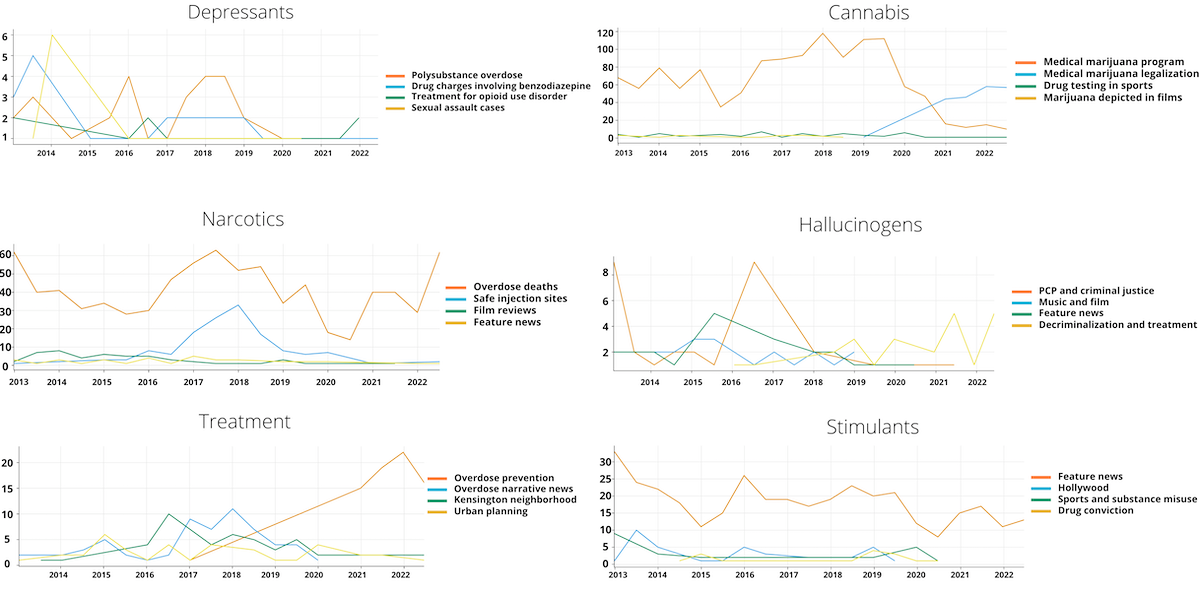
Topic evolution per class of drug per year. PCP: phencyclidine.

Our analysis revealed distinct thematic patterns across different drug classes (Figure 2). For hallucinogen-related coverage, we identified 4 main topics: phencyclidine (PCP) and criminal justice, music and film, feature news (eg, a human-interest story on how a local who experiences posttraumatic stress disorder [PTSD] uses lysergic acid diethylamide to treat their symptoms [48]), and decriminalization and treatment. Criminal justice coverage dominated early discussions in 2013 but showed a marked decline until a substantial spike in 2017. Feature news emerged as a consistent theme throughout the period, peaking in 2016, while coverage of hallucinogens in music and film maintained a relatively stable presence. Notably, discussions of decriminalization and treatment gained increasing prominence from 2019 onward, becoming a dominant theme by 2022.

Articles discussing depressants centered on 4 key themes: polysubstance overdose, drug charges involving benzodiazepine, treatment for OUD, and sexual assault cases. Coverage of sexual assault cases saw a dramatic spike in 2014 before steadily declining, while reporting on polysubstance overdose showed cyclical patterns with peaks in 2016 and 2018 to 2019. Benzodiazepine-related charges received heightened attention in early years (peaking in 2014) but diminished over time, while treatment discussions remained relatively steady before showing renewed interest in 2022.

Cannabis coverage demonstrated the most dramatic thematic shifts over the decade. The 4 main topics identified were the medical marijuana program, medical marijuana legalization, drug testing in sports, and marijuana depicted in films. Coverage of the medical marijuana program dominated from 2013 to 2020, peaking in 2018 with nearly 96% (120/125) of articles. However, a significant transition occurred around 2020, when legalization discussions began to overtake program-related coverage. By 2022, legalization had become the primary focus, with about 75% (60/75) of articles, while program-related coverage declined to approximately 20% (15/75) of articles. Sport-related drug testing and film depictions maintained a minimal but consistent presence throughout the period, rarely exceeding >5 articles per year.

Among articles that mentioned narcotics, we observed the following top 4 themes: overdose deaths, safe injection sites, film reviews, and feature news. Coverage around overdose deaths within the city consistently received the most coverage throughout the decade, starting at around 85% (60/70) of the articles in 2013, declining to approximately 66% (30/45) of articles through the mid-2010s, before showing significant spikes in 2018 (>60 articles) and 2022 (>60 articles). Coverage of safe injection sites emerged as a prominent theme around 2017, peaking in 2018 with >30 articles, before gradually declining through 2022. Film reviews (articles discussing a film where narcotics are present) and feature news maintained relatively low but steady coverage, rarely exceeding 5 articles per year.

Stimulant-related coverage revealed 4 distinct themes: feature news, Hollywood, sports and substance misuse, and drug conviction. Feature news dominated the coverage, with higher frequency in earlier years (>30 articles in 2013) compared to later years (approximately 15/15, 100% in 2022), with notable fluctuations including a peak of 80% (25/30) of articles in 2016. Coverage related to Hollywood and sports showed higher prominence in earlier years (2013 to 2014) but maintained minimal presence afterward. Drug conviction coverage remained consistently low throughout the period, with slight increases in 2015 and 2019.

Treatment-related coverage demonstrated evolving priorities through overdose prevention, overdose narrative news, the Kensington neighborhood, and urban planning. Most notably, coverage of overdose prevention showed a dramatic increase from 2019 onward, reaching >20 articles by 2021. Overdose narrative news peaked around 2018 to 2019 with about 10 articles per year, while coverage of the Kensington neighborhood showed heightened attention in 2017 (10/15, 66% of articles). Urban planning maintained relatively consistent but low coverage throughout the period.

These patterns highlight how coverage of different drug classes responded to evolving public health concerns and policy initiatives. While narcotics coverage maintained a consistent focus on overdose deaths, treatment coverage showed a clear shift toward prevention strategies in recent years. Meanwhile, stimulant coverage demonstrated a broader focus on social and cultural aspects rather than public health concerns.

### Sentiment Pattern and Aspect Analysis

**Figure 3:**
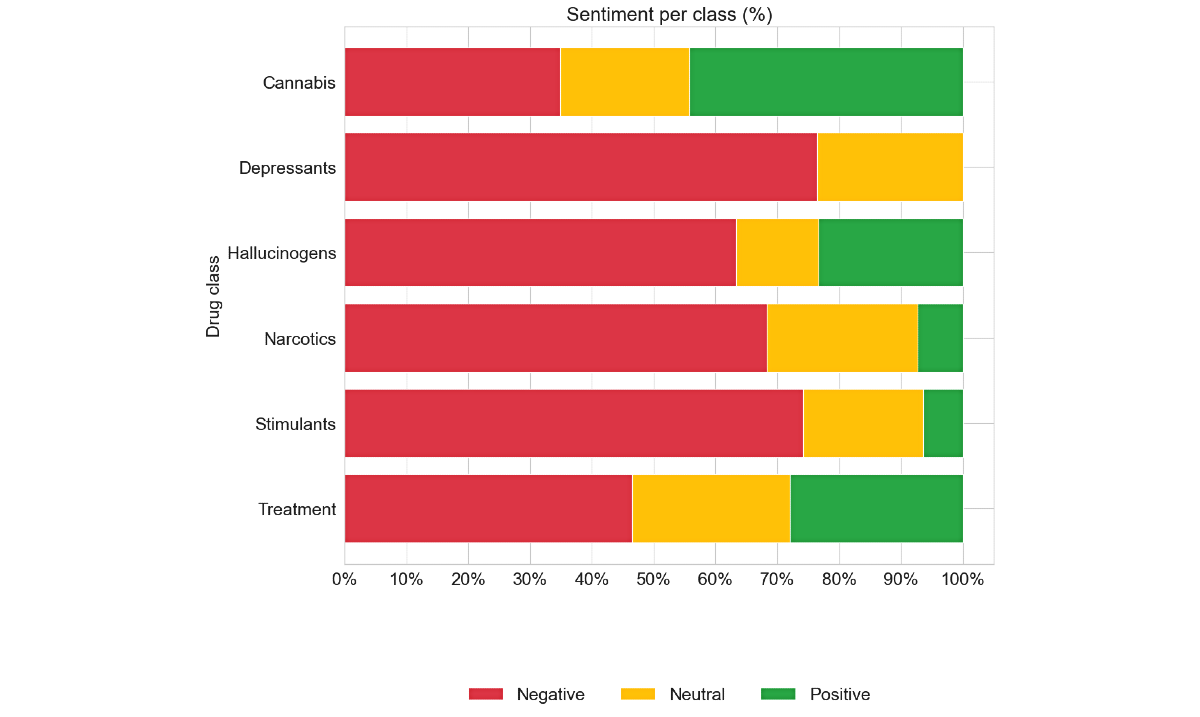
Proportion of aspects with positive, negative, and neutral sentiments per class.

Our sentiment analysis revealed distinct patterns across 3 dimensions: overall sentiment distribution by drug class, temporal changes in sentiment, and evolution of thematic aspect groups over time. The overall sentiment distribution (Figure 3) showed cannabis and hallucinogens having the highest proportion of positive aspects, while depressants and narcotics demonstrated predominantly negative coverage. Treatment-related coverage showed a more balanced distribution between positive and negative sentiments, reflecting the complex nature of intervention discussions.

**Figure 4:**
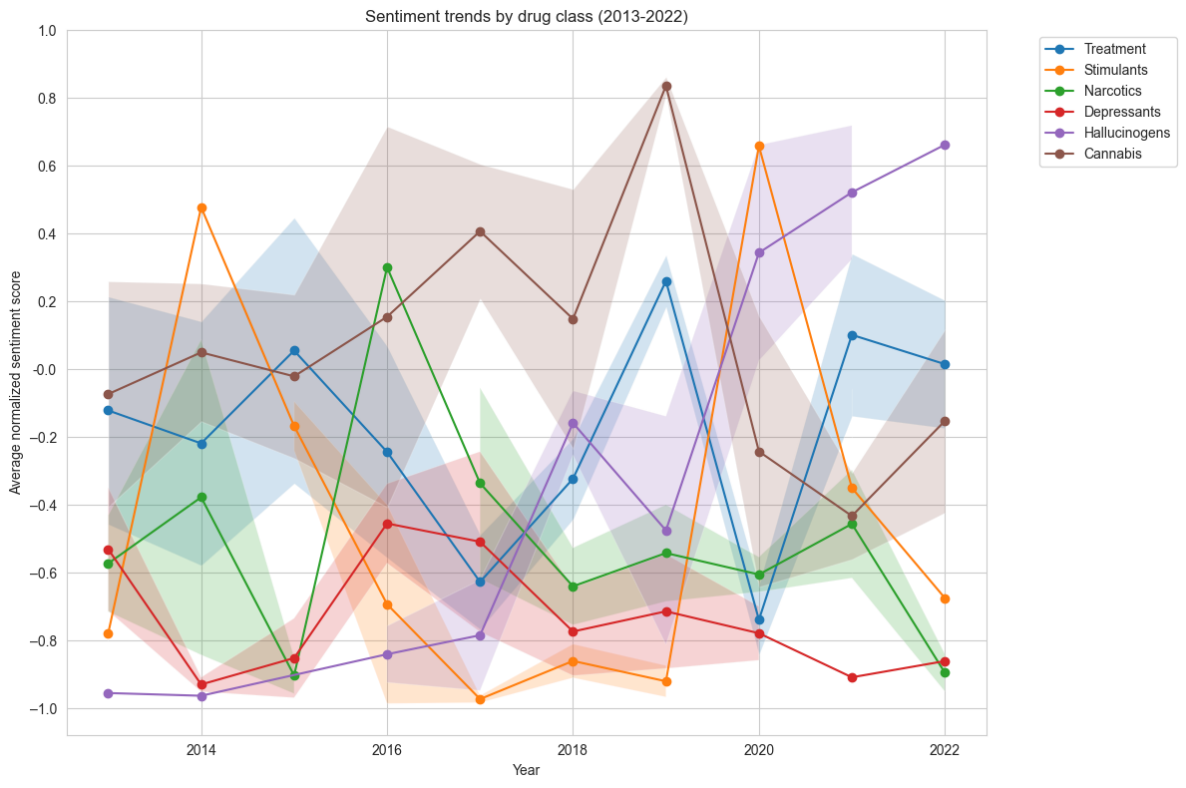
Average sentiment scores for each drug class from 2013 to 2022. Sentiment scores are normalized to range from –1 (most negative) to 1 (most positive), with 0 representing neutral sentiment. Shaded areas represent CI.

Temporal sentiment patterns (Figure 4) revealed significant evolution in coverage tone across drug classes. Hallucinogens showed the most dramatic positive shift, moving from strongly negative coverage in 2013 to 2014 to increasingly positive coverage by 2022. Cannabis coverage demonstrated considerable volatility but maintained generally neutral to positive sentiment, while depressants consistently received the most negative coverage throughout the period.

**Figure 5:**
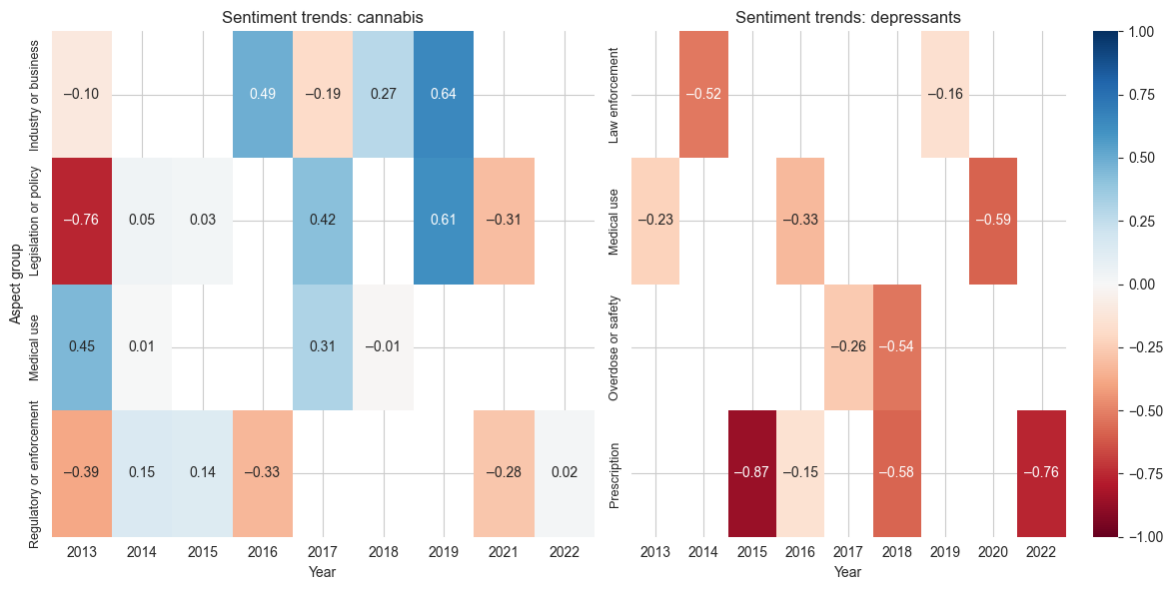
Sentiment scores across aspect groups for the drug classes cannabis and depressants. Values represent average sentiment scores ranging from –1 (most negative: red) to 1 (most positive: blue), with white indicating neutral sentiment.

Analysis of thematic aspect groups over time (Table 3) provided deeper insight into these sentiment patterns (Figure 5). To facilitate interpretation and discussion, we grouped aspects into thematic categories within each drug class based on semantic similarity. As shown in Figure 5, for cannabis, industry and business aspects showed strong positive sentiment score (SS) peaks in 2016 (0.49) and 2019 (0.64), while legislation and policy aspects evolved from highly negative (–0.76 in 2013) to positive (0.61 in 2019) sentiment scores before declining again (–0.31 in 2022). Medical use maintained a relatively stable, slightly positive sentiment, while regulatory and enforcement aspects remained predominantly negative.

**Table 3:** Aspect groupings by drug class.

Drug class and aspect group		Representative aspects
**Cannabis**		
	Legislation or policy	marijuana legalization, cannabis legislation, marijuana reform, marijuana laws
	Medical use	medical marijuana, medical cannabis, medicinal marijuana, medical marijuana program
	Industry or business	marijuana dispensaries, cannabis industry, cannabis investments, nascent marijuana industry
	Regulatory or enforcement	marijuana convictions, marijuana offenses, recreational marijuana, cannabis impairment
**Treatment**		
	Overdose prevention	naloxone spray, narcan, overdose drug, lifesaving drug, reversal medication
	Opioid treatment	buprenorphine prescription, methadone programs, buprenorphine program, prescribing buprenorphine
	Crisis response	overdose fatalities, drug costs, opioid crisis, opioid epidemic, drug response
	Medical or clinical	opioid painkillers, opioid prescriptions, prescription drugs, opioid use disorder
**Narcotics**		
	Overdose	overdose crisis, overdose victims, fatal overdose, overdose deaths, fentanyl overdoses
	Law enforcement	drug possession, federal drug laws, drug convictions, border protection, drug trafficking
	Public health	opioid epidemic, aids crisis, public drug use, neonatal abstinence syndrome
	Policy or treatment	new jersey drug policy alliance, opioid litigation, drug laws ease, opioid drugmakers
**Hallucinogens**		
	Medical or therapeutic	new psychedelic therapies, psychedelic therapeutics company, new psychiatric medicines
	Law enforcement	drug charges, drug arrests, smuggled drugs, illegal psychedelic drugs
	Substances	lsd, cannabis, marijuana, psychoactive compounds, crack cocaine
	Social context	party drug, prevalent drug use, favorite drug conversation
**Depressants**		
	Medical use	sleep drug zolpidem, insomnia drugs, medical marijuana, lorazepam, using ambien
	Overdose or safety	xanax overdose, overdose deaths, overdose statistics, opioid overdoses
	Prescription	prescription opioids, benzodiazepines, morphine, zaleplon prescription
	Law enforcement	homicide case, county district attorney, montgomery county prosecutors
**Stimulants**		
	Law enforcement	drug violations, cocaine possession, large cocaine distribution ring, drug trafficking
	Health or treatment	addiction treatment program, few medication options, antidepressant, methamphetamine use
	Crime related	crime spree, customs officials, probation violation
	Substance specific	illicit fentanyl, cocaine use, lehtera cocaine, crack cocaine buyer, largest cocaine seizure

The evolution of cannabis-related sentiment is particularly evident in key legislative and policy aspects (Table 4). Coverage of recreational marijuana use and decriminalization shifted from predominantly negative sentiment in 2013 (with quotes expressing opposition to decriminalization) to increasingly positive coverage by 2019, when discussions focused on potential economic and social benefits. Similarly, marijuana legalization aspects evolved from skeptical coverage in 2013 to 2014 to more supportive framing by 2019, although this positive sentiment declined in later years as implementation challenges emerged. This transformation in coverage tone reflects broader societal shifts in cannabis policy discourse over the decade. This evolution in cannabis coverage is further reflected in the terminology used, where aspects associated with “marijuana” often carried negative connotations, while “cannabis” was framed more positively. For example, the Marijuana Opportunity Reinvestment and Expungement Act’s discussion in 2019 used the term “cannabis” when describing positive policy developments (Marijuana Opportunity Reinvestment and Expungement Act of 2019 [49]), while earlier negative coverage of decriminalization by New Jersey Governor Christie used “marijuana” (Inquirer Staff, June 18, 2015, NEW JERSEY: Sharp increase in backing for legalizing marijuana, the Philadelphia Inquirer; Multimedia Appendix 1). Within the cannabis class, “medicinal marijuana” consistently received more positive coverage than “recreational use.”

Depressants showed consistently negative sentiment across all aspect groups, with prescription-related aspects showing particularly negative sentiment (–0.87 in 2015 and –0.76 in 2022). Law enforcement aspects improved slightly from –0.52 in 2014 to –0.16 in 2019, while medical use aspects grew increasingly negative over time, reaching –0.59 by 2020 (Figure 5). The overdose or safety category emerged with negative sentiment (–0.26 to –0.54) in later years, reflecting growing concerns about safety risks. The consistently negative coverage of depressants was particularly evident in discussions of combined drug use, especially regarding benzodiazepines with opioids and their associated overdose risks. The sentiment toward “insomnia drugs” notably deteriorated between 2013 and 2016, influenced by growing concerns about prescription policies and pharmaceutical companies’ transparency in warning labels (Multimedia Appendix 1).

**Figure 6:**
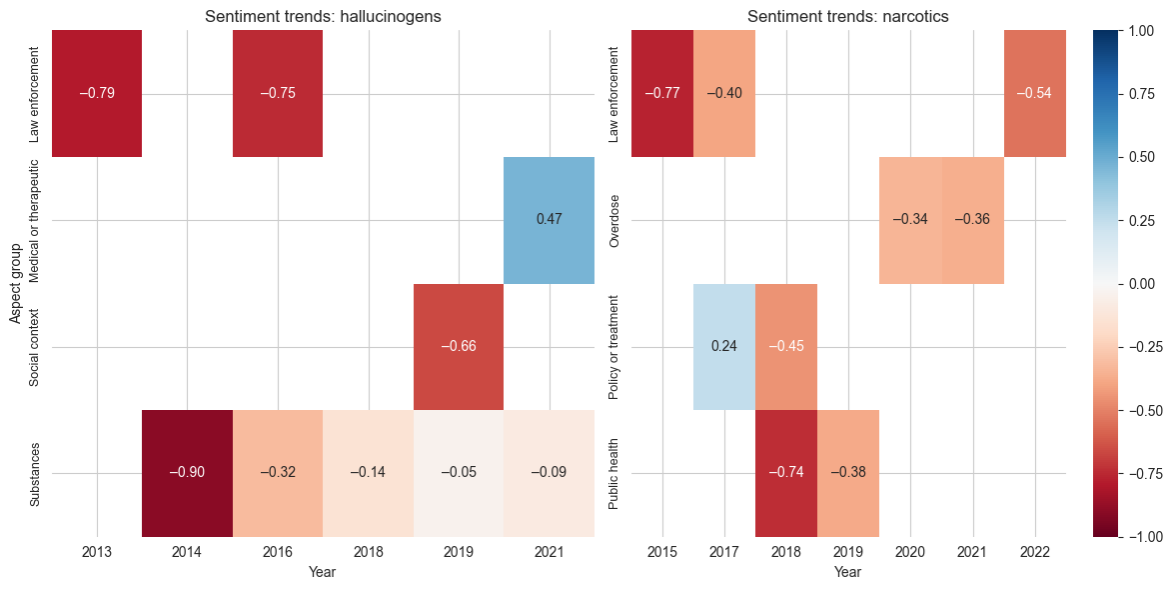
Sentiment scores across aspect groups for the drug classes hallucinogens and narcotics. Values represent average sentiment scores ranging from –1 (most negative: red) to 1 (most positive: blue), with white indicating neutral sentiment.

Hallucinogen coverage showed the most dramatic thematic evolution (Figure 6). While law enforcement aspects maintained strong negative sentiment (–0.79 in 2013 and –0.75 in 2016), medical or therapeutic aspects emerged in later years with notably positive sentiment (0.47 in 2021). As detailed in Multimedia Appendix 1, this shift is particularly evident in the coverage transition from negative associations with crack cocaine (2014) and cannabis (2018) to increasingly positive framing of medicinal cannabis (2019) and new psychedelic therapies (2021). This positive shift in medical or therapeutic aspects may be attributed to recent studies on psychedelic drugs for treating mental health disorders, such as treatment-resistant depression and PTSD. Substance-specific aspects showed gradual improvement from highly negative sentiment (–0.90 in 2014) to slightly negative sentiment (–0.09 in 2021), while social context aspects appeared sporadically with negative sentiment (–0.66 in 2019). Table 5 provides an exemplar excerpt from The Philadelphia Inquirer that reflects this evolution, particularly the positive sentiment regarding the therapeutic potential of hallucinogenic drugs for treating PTSD.

Building on these contrasting patterns, narcotics coverage maintained consistently negative sentiment across all aspect groups (Figure 6). Law enforcement aspects showed persistent negative sentiment, although improving slightly from –0.77 in 2015 to –0.54 in 2022. The overdose aspect group emerged in later years with moderately negative sentiment (–0.34 in 2020 and –0.36 in 2021), while public health aspects improved from –0.74 in 2018 to –0.38 in 2019. Policy or treatment aspects showed brief positive sentiment (0.24 in 2017) before turning negative (–0.45 in 2018), reflecting the challenges in implementing treatment initiatives.

**Figure 7:**
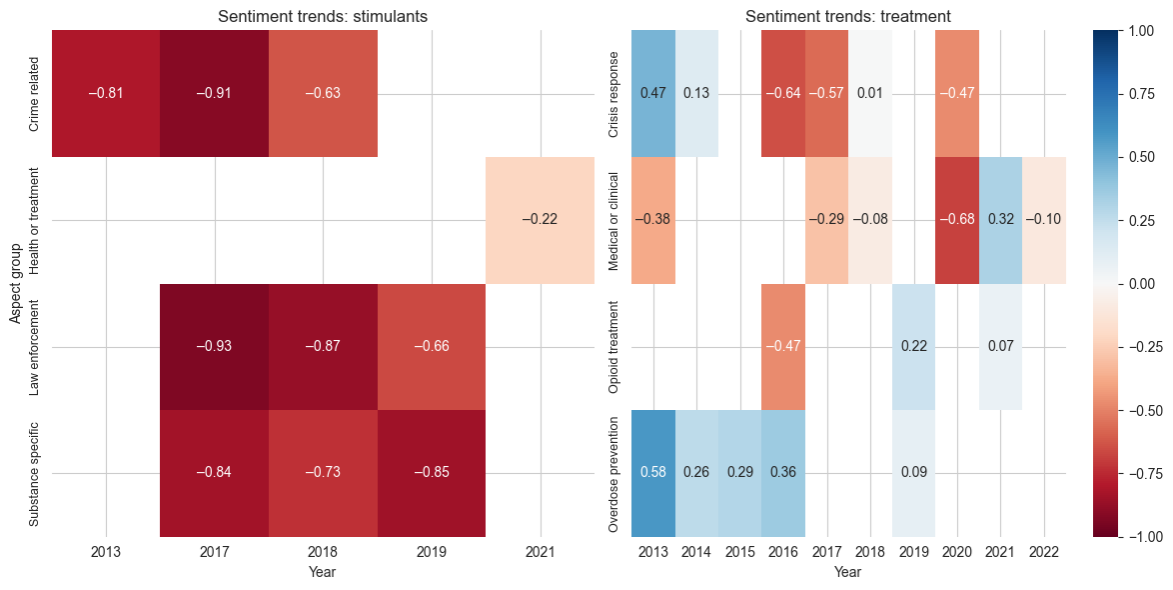
Sentiment scores across aspect groups for the drug classes stimulants and treatment. Values represent average sentiment scores ranging from –1 (most negative: red) to 1 (most positive: blue), with white indicating neutral sentiment.

Similarly negative but with even stronger intensity, stimulant coverage demonstrated some of the most consistently negative sentiment patterns across all drug classes (Figure 7). Crime-related aspects maintained strongly negative sentiment throughout (–0.81 in 2013, –0.91 in 2017, and –0.63 in 2018), while law enforcement aspects showed similarly negative shifts (–0.93 to –0.66 between 2017 and 2019). Substance-specific coverage remained consistently negative (–0.84 to –0.85), although health or treatment aspects emerged in later years with slightly less negative sentiment (–0.22 in 2021).

**Table 4:** Changes of tone toward marijuana decriminalization.

Article	Aspect	Year	Sentiment
“...Craig T. Steckler, president of the police officers’ conference, led his introduction of holder by criticizing the justice department’s decision this year not to challenge state laws in Colorado and Washington that allow recreational marijuana use^a^...”	Recreational marijuana use	2013	Negative
“...One question he answered: he said that he disagreed with U.S. Attorney General Eric Holder’s memo Thursday saying the Obama administration would not challenge the new marijuana decriminalization^a^ laws in Colorado and Washington. ‘I think it’s a mistake for him to turn his back and essentially by fiat legalize marijuana in Colorado and Washington,’ Christie said...”	Marijuana decriminalization	2013	Negative
“...New Jersey is one of 23 states that have legalized medical marijuana despite a longtime federal prohibition against selling or using the drug for medical or recreational reasons...‘this federal policy toward state-level marijuana legalization efforts^b^ creates a situation in which the medical marijuana industry is in existence, integrating into local, state, and national economies, and employing thousands of people, some of whom are represented by labor unions or involved in labor organizing efforts despite the industry’s illegality,’ the opinion said...”	Marijuana legalization efforts	2015	Neutral
“...marijuana is a schedule 1 drug, which means the federal government treats it as if it were as dangerous as heroin or lsd and has no medical benefit. The act would also require authorities to remove federal cannabis convictions from millions of criminal records. More than two-thirds of American voters support full [marijuana] legalization^c^, according to poll results released Nov. 14 by the Pew Research Center... ‘It’s the first piece of marijuana cannabis legislation^c^ in Congress to move this far. And this could lead to more local reform as well,’ said Goldstein, a South Jersey-based organizer for the National Organization for the Reform of Marijuana Laws.”	Marijuana cannabis legislation	2019	Positive

^a^Negative aspects.

^b^Neutral aspects.

^c^Positive aspects.

**Table 5:** Example aspects and excerpts of article text with sentiment: “+” denotes a positive sentiment and “–” denotes a negative sentiment.

Type of drugs and aspect; year		Sentiment	Excerpt
**Hallucinogens**			
	“New psychiatric medicines”; 2021	+	“...last summer, the University of North Carolina received nearly $27 million from DARPA, the research arm of the U.S. Department of Defense, to develop new psychiatric medicines from psychedelics...Hopkins, along with New York University, recently completed phase 3 clinical trials with the nonprofit multidisciplinary association for psychedelic studies for MDMA-assisted therapy for PTSD...”
**Narcotics**			
	“Medical marijuana”; 2017	+	“...he said. marijuana helped him with sobriety, and also helped him get off oxycodone and several other medications he was prescribed over the years for the pain caused by his many parachuting missions and the anxiety that PTSD triggered, he said. Karpowich said he participated in at least a dozen protests at the statehouse in Trenton, holding signs and ‘ambushing lawmakers’ to get them to consider expanding the medical marijuana program. ‘veterans have served their country, and no one should tell them they can’t use marijuana if it helps them,’...”
	“Border protection”; 2022	−	“...he unleashed the worst border crisis in U.S. history. u.s. customs and border protection reported more than 1.7 million encounters with illegal migrants at the southern border, nearly four times the number the year before, the highest annual total on record—including 378,000 who were not from Mexico, Honduras, El Salvador, or Guatemala. seizures of deadly fentanyl more than doubled in 2021, and is closely connected to a surge in overdose deaths, which reached a historic high.”
**Treatment**			
	“Drug costs”; 2016	−	“...basic lifesaving medicines that emergency workers use every day are getting so costly, officials are scrambling to figure out how to pay for them. and as patients struggle with drug costs, EMS workers and emergency room doctors are seeing the impact. the price paid by Philadelphia emergency medical services for naloxone, which reverses opioid overdoses, has risen 150 percent since 2013...the opioid epidemic rages and record numbers of people die of overdoses, the cost of generic naloxone has more than doubled...”

In contrast to these predominantly negative patterns, treatment coverage showed the most varied sentiment patterns, with distinct patterns across different aspect groups (Figure 6). Overdose prevention maintained a positive sentiment in the early years (0.58 in 2013, declining to 0.09 by 2019), while crisis response fluctuated dramatically, shifting from positive sentiment (0.47 in 2013) to negative (–0.64 and –0.57 in 2016 and 2017, respectively), back to neutral (0.01 in 2018), and then negative again (–0.47 in 2020). Medical or clinical aspects showed generally negative sentiment but with notable improvement in 2021 (0.32) before declining again (–0.10) in 2022. Opioid treatment aspects emerged with negative sentiment (–0.47 in 2016) but showed modest improvement in later years (0.22 in 2019 and 0.07 in 2022), suggesting growing acceptance of medication-assisted treatment approaches. The evolution of treatment coverage is particularly evident in the changing focus on positive aspects over time. Naloxone-related aspects (described as a “life-saving drug”) dominated positive coverage from 2013 to 2016, while buprenorphine prescription emerged as a central positive aspect from 2019 to 2021, before shifting to negative coverage due to concerns about misuse and street value (Multimedia Appendix 2). Cost-related aspects, particularly regarding naloxone prices and their impact on emergency medical service workers, contributed to negative sentiment spikes in 2016 (Table 5).

These temporal and thematic patterns reveal distinct evolutionary trajectories in media coverage of different substances and treatments. While some substances, particularly stimulants and depressants, maintained consistently negative coverage focused on crime and risk, others showed notable transitions. Hallucinogens evolved toward increasingly positive coverage centered on therapeutic potential, while cannabis coverage reflected complex societal debates around legalization and medical use. Treatment-related coverage, although variable, suggested growing acceptance of evidence-based interventions despite persistent challenges in implementation.

## Discussion

### Principal Findings

This study aimed to identify narrative shifts, including topics and sentiment, of drug news reporting within The Philadelphia Inquirer over a 10-year period. By grouping the articles based on drug classifications set by the National Institute on Drug Abuse and using co-occurrence analysis, dynamic topic modeling, and sentiment analysis, we uncovered several key findings. The co-occurrence analysis identified an expected connection between opiate narcotics (heroin and fentanyl) and substances such as cannabinoids or methadone, which are commonly used to alleviate opioid dependency symptoms, such as cravings and withdrawal severity. These findings align with provisional drug use and rise in overdose reported both nationwide [50] and within Philadelphia [51]. The emergence of “cocaine” comentions in 2018 matches the growing concern of opiate or stimulant co-use trends [52], as unintentional overdose deaths often involve the combined use of fentanyl or a stimulant such as cocaine. Buprenorphine, a safe and effective treatment for OUD, emerged in the coverage in 2020, with the most mentions (17 articles that mention buprenorphine in 2022 divided by 94 articles mentioning buprenorphine total, 18.1%) in 2022, likely correlated to policy changes making it more accessible [53].

The dynamic topic modeling revealed a strong focus on overdose deaths in the narcotics class. While we do not find evidence of sensationalism reported in other media studies [7,18,19,54], our analysis revealed a persistent focus on overdose deaths in narcotics coverage. Beyond direct event reporting, we observed substantial coverage of drug portrayals in entertainment media [55,56], suggesting news media’s dual role in both reporting and reflecting on drug-related narratives.

Sentiment analysis revealed distinct trajectories for different substances. Cannabis coverage showed a marked evolution from negative to predominantly positive sentiment, particularly around medical applications and legalization. This shift coincided with significant local developments, including public figures’ advocacy [57,58] and institutional initiatives [59]. In contrast, depressants and narcotics maintained consistently negative coverage, focusing on risks and criminal justice aspects. Hallucinogens showed the most dramatic positive shift, driven by emerging therapeutic applications, while treatment coverage reflected the complex challenges of implementing harm reduction approaches. These findings highlight the evolving nature of drug-related news coverage and its potential to shape public perceptions and policy discussions, as elaborated in the Implications section.

### Implications

These findings have several important implications for both journalism practice and drug policy. Research has established that news media plays a significant role in shaping public perceptions and attitudes [1-5]. Through our analysis of The Philadelphia Inquirer's drug coverage, we track how these potentially influential media narratives have evolved over time. The evolution of cannabis coverage, characterized by a shift from negative to predominantly positive sentiment, particularly regarding medical applications and legalization, demonstrates how media reporting reflects shifting narratives around drug use.

The shift in cannabis coverage coincided with several significant local events in 2018: the admission of cannabis use by a former Philadelphia Eagles player [57], advocacy for recreational legalization by Mayor Jim Kenney [58], and the inaugural Cannabis Opportunity Conference in Philadelphia [59]. These events and the media’s increasingly positive coverage of them exemplify the interplay between social change, policy developments, and media narratives.

Conversely, the persistent negative framing of certain substances, particularly in narcotics coverage (eg, “overdose crisis,” “overdose victims,” and “public drug use”), potentially reinforces stigma that can hinder public health approaches. The rare positive aspects (eg, the “New Jersey Drug Policy Alliance”; Multimedia Appendix 2) in narcotics coverage emerged only in relation to harm reduction initiatives, highlighting the potential challenge of incorporating harm reduction narratives into mainstream media discourse. While fear-based narratives can contribute to moral panic [60], our analysis suggests that balanced reporting can help normalize evidence-based interventions, as seen in the evolving coverage of cannabis and psychedelic therapies [61-63].

The pattern of coverage evolution raises important questions about the media’s role in normalizing certain substances [64] while potentially stigmatizing others [65,66]. While cannabis coverage has grown increasingly nuanced and accepting, particularly regarding medical applications, coverage of narcotics remains predominantly negative, often emphasizing criminal justice aspects rather than public health approaches. News outlets have the potential to influence public discourse by providing more balanced coverage that includes harm reduction perspectives while maintaining responsible reporting on public health concerns.

Furthermore, our study found an increasing discussion of naloxone, an overdose prevention medication, and buprenorphine, a safe and effective treatment for OUD. Similar to previous work [67], the increasing coverage of these treatments suggests that news outlets can help to educate the public about evidence-based approaches to drug treatment and policy, which is important in the context of the ongoing opioid overdose crisis [67].

Finally, news outlets can impact policy by reporting on the work being done to reduce harm versus criminality aspects of drug use [17,66,68]. Our findings suggest that shifting news coverage from focusing on law enforcement to emphasizing prevention-oriented solutions may change the public’s and nonexpert policy makers’ views on drug issues, shifting from blame to more health-focused and harm reduction perspectives [22]. This shift mirrors a broader historical trajectory: while the stigmatization of drug users in the United States historically resulted in punitive policies [69], recent years have seen movement toward public health–oriented policies, such as expanding treatment and supporting overdose prevention laws [70].

However, our analyses reveal that discussions on harm reduction are still limited and have not been a prominent aspect of drug-related news in the past decade. As media coverage continues to evolve, it is hoped that future coverage will increasingly focus on compassionate and effective harm reduction strategies for affected communities and people who use drugs. By avoiding fear-based strategies and promoting balanced narratives, the media can greatly contribute to fostering a more empathetic and informed public discourse on drug use, ultimately influencing policy decisions and reducing harm in affected communities [65,66].

### Limitations

Our findings should be interpreted in the context of several limitations. First, our analysis relies on articles from ProQuest’s database of The Philadelphia Inquirer, which may not represent the complete set of articles published during this period. This limitation stems from potential gaps in digital archiving and database coverage. Future studies could incorporate multiple database sources or direct newspaper archives to ensure comprehensive coverage.

Second, our study focused solely on news articles published in The Philadelphia Inquirer, and therefore, our findings are specific to this source and its coverage of substance use within Philadelphia and the United States. While this focus provides valuable insight into local media coverage in a city significantly impacted by drug use, it limits generalizability to other regions or media outlets. Further research is needed to explore how the portrayal of substance use in the media varies across different regions and cultures.

Third, while previous research has established connections between news media coverage and public attitudes, our analysis of news did not enable us to evaluate the impact of this news exposure on public opinion regarding the issues. Future studies could combine media content analysis with public opinion surveys to directly measure these relationships.

Finally, the findings of our study do not correlate or imply popularity in use. As shown in Table 1, *designer drugs* and *drugs of concern* are mentioned in only 16 (0.43%) and 11 (0.3%) of the 3661 articles, respectively. Owing to the sparse data on these topics in our corpus, we excluded them from further analysis. Future work could specifically target coverage of emerging substances to better understand their media representation.

### Conclusions

This study aimed to explore how The Philadelphia Inquirer, a prominent local newspaper covering Philadelphia, a city known nationwide for its ongoing drug crises, mentions commonly abused substances and how the narrativity and sentiment around these drugs change over time. To do so, we analyzed news articles published in The Philadelphia Inquirer between 2013 and 2022, using co-occurrence analysis, dynamic topic modeling, and aspect-based sentiment analysis, and found that cannabis was the most frequently discussed drug class, followed by narcotics. Aspects reported with hallucinogenic drugs tended to have a more positive tone compared to other categories of drugs, while articles on narcotics were the most negative. We also observed a significant focus on overdose and death-related aspects, but there was a noticeable lack of coverage related to harm reduction principles. This study highlights the linguistic shifts reported across various drug classes. It provides compelling evidence of the influence that news media outlets have in shaping discourse around drug use. This, in turn, contributes to creating a more informed and compassionate society, ultimately reducing the harm associated with drug use.

## Appendix

Multimedia Appendix 1 Representative aspects and article excerpts demonstrating sentiment shifts in media coverage of cannabis, hallucinogens, depressants, and treatment drugs (2013-2022). Color coding indicates negative (pink), neutral (yellow), or positive (green) sentiment, highlighting evolving media discourse around key drug-related topics.

Multimedia Appendix 2 Complete list of aspects (N=222) extracted from articles across all drug classes, showing neutral, positive, negative, and overall sentiment scores for each aspect (2013-2022).

## Abbreviations

BERT: bidirectional encoder representations from transformers
BERTopic: bidirectional encoder representations from transformers topic modeling technique
OUD: opioid use disorder
PTSD: posttraumatic stress disorder
RQ: research question
SS: sentiment score
SUD: substance use disorder

## Data Availability

The metadata of our dataset, codes, and results of this study are available in the Drugs in Inquirer repository [71].
