# Supplementary material for: Shifting Narratives in Media Coverage Across a Decade of Drug Discourse in the Philadelphia Inquirer: Qualitative Sentiment Analysis
Source: JMIR Infodemiology. 2025 May 13;5:e56004. doi: 10.2196/56004 (PMC12117267; doi:10.2196/56004)
Supplement: Multimedia Appendix 1 [file infodemiology_v5i1e56004_app1.docx]

Representative aspects and article excerpts demonstrating sentiment shifts in media coverage of cannabis, hallucinogens, depressants, and treatment drugs (2013-2022). Color coding indicates negative (pink), neutral (yellow), or positive (green) sentiment, highlighting evolving media discourse around key drug-related topics.

| **Class** | **Aspect** | **Article** | **Sentiment** | **Year** |
| --- | --- | --- | --- | --- |
| Cannabis | [new] marijuana laws | “... Powell coauthored the report, “do medical marijuana laws reduce addictions and deaths related to pain killers,” along with researchers from the University of California-Irvine, and the National Bureau of Economic research in Cam- bridge, mass. The study suggests that patients will substitute medical marijuana for addictive prescription opioids if cannabis products are available through dispensaries. between 2000 and 2010, opioid-related fatalities and reductions in treatment admissions in states with flourishing dispensaries declined by about 20 percent...” | Positive | 2018 |
|  |  | “... police could also confiscate your marijuana, says Chris Goldstein, an activist and regional organizer for the National Organization for the reform of Marijuana laws. however, DeVaughn Ward, senior legislative counsel for the marijuana policy project, says they’re not supposed to, because the law doesn’t “give [police] the ability to search or seize your  cannabis.” the state’s new marijuana laws also let local municipalities pass their own marijuana ordinances...” | Negative | 2021 |
|  | Marijuana decriminalization legalization | “... One question he answered: he said that he disagreed with U.S. Attorney General Eric Holder’s memo Thursday saying the Obama administration would not challenge the new marijuana decriminalization laws in Colorado and Washington. “I think it’s a mistake for him to turn his back and essentially by fiat legalize marijuana in Colorado and  Washington,” Christie said...” | Negative | 2013 |
|  |  | “... marijuana is a schedule 1 drug, which means the federal government treats it as if it were as dangerous as heroin or lsd and has no medical benefit. The act would also require authorities to remove federal cannabis convictions from millions of criminal records. More than two-thirds of American voters support full [marijuana] legalization, according to poll results released Nov. 14 by the Pew Research Center. The share of U.S. adults who oppose legalization has fallen from 52% in 2010 to 32%. The bill has the support of several Philadelphia-area members of Congress...” | Positive | 2019 |
|  | Medical marijuana | “... physicians statewide are also torn over the propriety of medical marijuana. Michael Fraser, executive director of the Pennsylvania Medical Society, testified that the doctors’ group does not recommend marijuana for medical use, but supports additional research into its effectiveness. “We believe a compelling case exists for a serious scientific examination of the potential medical use of marijuana,” said Fraser, but he added, “the legalization of marijuana for medical use is premature and unwise...” | Negative | 2014 |
|  |  | “...for the first time in the history of the seven-year-old New Jersey medical marijuana program, a health department panel will consider adding chronic pain and other ailments to the list of about a dozen conditions that qualify a patient to use cannabis in the state. “I think cannabis can replace all three of the medications I take for anxiety, migraines, and chronic pain,” said Bob Kane, 56, a retired landscaper from Ocean View...” | Positive | 2017 |
| Hallucinogens | Psychedelic drugs | “...doctors and patients are awaiting the Food and Drug Administration’s decision on another type of electrical stimulation that targets the vagus nerve. A trial that will for the first time scientifically test whether magic mushrooms really do help - no small feat in the risk-averse research world - may start soon. the question of whether illegal psychedelic drugs can help headache patients has been “radioactive,” said Brian Mcgeeney, a Boston medical center neurologist who thinks the drugs deserve more study...” | Negative | 2016 |
|  |  | “...People are just really excited,” said McLane, 40, who wants Philadelphia to be a leader in the field of psychedelic medicine ...MDMA helps people with PTSD, backers say, because it allows people to tunnel into their traumatic memories, accept them, and then learn to live with them...” | Positive | 2021 |
| Depressants | Opioid painkillers | “...Women, in general, are more likely to pursue health care and they are more likely to be prescribed these therapies” opioid painkillers - “for a medical reason,” said Cynthia Reilly, a pharmacist, and director of the Pew Charitable Trusts’ prescription drug abuse project. She said that women also are more likely to be prescribed benzodiazepines like valium, which make overdoses more likely when used in combination with opioids...” | Negative | 2016 |
|  |  | “...we owe them safe spaces the result is overdose deaths on a previously unimagined scale, all because we decided that opioid use — unlike alcohol, cigarettes, valium, and other substances used to cope with the world — was unacceptable. while we should do everything to help those who compulsively use, we shouldn’t simultaneously push users into the dangerous black market where each injection carries a substantial risk of death...” | Neutral | 2022 |
|  | Insomnia drugs | “...the FDA will now require that driving-simulation tests be conducted as part of any new application for insomnia drugs. IMS Health, a healthcare technology and information company with an office in Plymouth meeting, said that about 60 million prescriptions were written in 2011 for all sleeping pills, about two-thirds of those some form of zolpidem...” | Neutral | 2013 |
|  |  | “...but Whitcraft noted that incident occurred in September 2006, six months before Ambien and other makers of insomnia drugs were ordered to include a warning that the pills could leave patients susceptible to sleep-driving...” | Negative | 2016 |
| Treatment | Buprenorphine prescription | “...although any doctor with a drug enforcement administration license can prescribe the opioid painkillers that can spark addiction, federal law requires special training to prescribe a potential remedy. Sometimes, a bedside buprenorphine prescription can be enough to get patients started on treatment. it can ease patients’ withdrawal pain, so they are willing to hear from someone like O’Donnell, rather than leaving the hospital in search of drugs...” | Positive | 2019 |
|  |  | “...There are unscrupulous doctors who hand out prescriptions and pharmacists who fill them. Subutex, which con- sists of buprenorphine alone, is easier to misuse and typically has higher street value than suboxone, a combination of buprenorphine and the overdose-reversal drug naloxone. in the case against Njoku’s pharmacy, an assistant U.S. attorney explained that the DEA “got slapped hard for being asleep at the switch as the opioid crisis ramped up. they’re trying to make sure that Subutex doesn’t become the next problem,” according to court transcripts...” | Negative | 2021 |
|  | Drug costs | “...basic lifesaving medicines that emergency workers use every day are getting so costly, officials are scrambling to figure out how to pay for them. and as patients struggle with drug costs, workers and emergency room doctors are seeing the impact. the price paid by Philadelphia emergency medical services for naloxone, which reverses opioid overdoses, has risen 150 percent since 2013...” | Negative | 2016 |
|  |  | “…As the city's harm reduction coordinator, Herens regularly conducts Narcan training and works to get the overdose-reversing drug into more people's hands. (The city has encouraged every Philadelphian to carry it, the Pennsylvania surgeon general has signed a standing order so anyone can get it without a prescription, and insurers such as Independence Blue Cross are offering it to subscribers at no cost. In its generic form (naloxone), the drug costs about $20 to $40.) "Naloxone is pretty easy to get," Herens told Thomas and Prescott.” | Neutral | 2018 |
